# A pilot study for robot appearance preferences among high-functioning individuals with autism spectrum disorder: Implications for therapeutic use

**DOI:** 10.1371/journal.pone.0186581

**Published:** 2017-10-13

**Authors:** Hirokazu Kumazaki, Zachary Warren, Taro Muramatsu, Yuichiro Yoshikawa, Yoshio Matsumoto, Masutomo Miyao, Mitsuko Nakano, Sakae Mizushima, Yujin Wakita, Hiroshi Ishiguro, Masaru Mimura, Yoshio Minabe, Mitsuru Kikuchi

**Affiliations:** 1 Department of Clinical Research on Social Recognition and Memory, Research Center for Child Mental Development, Kanazawa University, Ishikawa, Japan; 2 Departments of Pediatrics, Psychiatry and Special Education, Vanderbilt Kennedy Center, Nashville, Tennessee, United States of America; 3 Department of Neuropsychiatry, Keio University School of Medicine, Tokyo, Japan; 4 Department of Systems Innovation, Graduate School of Engineering Science, Osaka University, Osaka, Japan; 5 JST ERATO ISHIGURO Symbiotic Human-Robot Interaction, Osaka, Japan; 6 Service Robotics Research Group, Intelligent Systems Institute, National Institute of Advanced Industrial Science and Technology, Ibaraki, Japan; 7 Department of Psychosocial Medicine, National Center for Child Health and Development, Tokyo, Japan; Tokyo Daigaku, JAPAN

## Abstract

Recent rapid technological advances have enabled robots to fulfill a variety of human-like functions, leading researchers to propose the use of such technology for the development and subsequent validation of interventions for individuals with autism spectrum disorder (ASD). Although a variety of robots have been proposed as possible therapeutic tools, the physical appearances of humanoid robots currently used in therapy with these patients are highly varied. Very little is known about how these varied designs are experienced by individuals with ASD. In this study, we systematically evaluated preferences regarding robot appearance in a group of 16 individuals with ASD (ages 10–17). Our data suggest that there may be important differences in preference for different types of robots that vary according to interaction type for individuals with ASD. Specifically, within our pilot sample, children with higher-levels of reported ASD symptomatology reported a preference for specific humanoid robots to those perceived as more mechanical or mascot-like. The findings of this pilot study suggest that preferences and reactions to robotic interactions may vary tremendously across individuals with ASD. Future work should evaluate how such differences may be systematically measured and potentially harnessed to facilitate meaningful interactive and intervention paradigms.

## Introduction

Autism spectrum disorder (ASD) is a neurodevelopmental disorder that affects the ability of an individual to communicate and understand social cues throughout their life. The social impairments associated with ASD can have a strong effect on the quality of interactions with other individuals. A variety of therapeutic and educational methods have been developed to assist individuals with ASD. Some of these programs have been moderately successful in symptom management, and in promoting increased interaction with others.

Recent rapid technological advances have enabled robots to fulfill a variety of human-like functions, leading researchers to use such technology for the development and subsequent validation of robotic interventions for individuals with ASD [1–3]. Indeed, some investigators have argued that recent robotic applications could be effectively harnessed to provide innovative clinical treatments for individuals with ASD [4–9], as many individuals with ASD have been shown to demonstrate a higher degree of task engagement when interacting with robots as compared to that with human adults [6, 9–12]. In fact, growing anecdotal evidence indicates that the use of robots may provide unique opportunities for assisting individuals with ASD [13].

Although a variety of robots have been proposed as possible therapeutic tools for interventions for individuals with ASD, the physical appearances of humanoid robots currently used in therapy with these patients are highly varied [13]. Accordingly, robot developers and therapists are interested in identifying the optimal appearance of robots used in interventions. They have recently attempted to examine preference for robot appearance in individuals with ASD.

For example, "KASPAR" [14–16] is a humanoid robot that has specific human-like features, but has been deliberately designed so that it is perceived as a robot. The face of the KASPAR robot can show a range of simplified expressions.

Our preliminary study shows that about 60% of children with typical development prefer interacting with a mechanical robot to an android robot. In addition, previous studies have shown that the general populace prefer robot-like robots to highly human-like androids [17]. When designing objects for use by people with ASD, researchers often subscribe to the notion that "simpler is better", i.e., individuals with ASD will gravitate toward simple, mechanical objects [13, 14, 18–20]. However, with the exception of the study by Robins, Dautenhahn, and Dubowski [18], few studies have investigated differences in visual preference in individuals with ASD with respect to the appearance of humanoid robots. Robins, Dautenhahn, and Dubowski et al. [18] evaluated the importance of robot appearance for individuals with ASD using a robot resembling an "ordinary man" (Theatrical Robot) and a robot that looked like a "pretty doll" (Robota). The Theatrical Robot was presented either as an ordinary human or with plain clothing and a featureless, masked face. Robota was presented either as a human-like "pretty doll" or as a "robot" with plain white clothes. In both instances, individuals with ASD appeared to prefer interacting with the robot that looked less human-like to the robot that looked more human-like. Thus, the authors concluded that robots meant to interact with individuals with ASD should be less detailed and less visually complex than humans, while still conforming to the humanoid form. These results are consistent with the notion shared by researchers that "simpler is better", i.e., individuals with ASD prefer simple, mechanical objects [13]. However, the study by Robins, Dautenhahn, and Dubowski et al. [18] comprised only four individuals with ASD, each twice exposed to the two conditions. Thus, the authors suggested that confirmatory studies be conducted to obtain more data on the responses of children to different robot appearances [18]. The appearance of humanoid robots is likely to be very important for designing tools that are efficacious in assisting individuals with ASD.

The ultimate goal of robot-assisted ASD therapy is the generalization of social skills obtained during the robot sessions to subsequent interactions with humans. For this purpose, robots that are more human-like may be advantageous as compared to mechanical robots. Therefore, the optimal appearance of robots used for ASD therapy should be located at some point on the Humanoid-Non-Humanoid spectrum, and it might be beneficial to vary this point according to the severity of ASD.

In the present study, we examined how appearance affects the humanoid robotic preferences of high-functioning individuals with ASD. We hypothesized that many high-functioning individuals with ASD, especially those with more severe autistic traits, would prefer plain, visibly mechanical robots over those with a more human-like appearance.

## Material and methods

### Participants

The current study was approved by the ethics committee of the National Center for Child Health and Development. Participants were recruited from the National Center for Child Health and Development. All procedures involving human participants were conducted in accordance with the ethical standards of the institutional and/or national research committee, and with the 1964 Helsinki Declaration and its later amendments or comparable ethical standards. After a complete explanation of the study, all participants and their parents provided written, informed consent. All participants and their parents agreed to participate in the study. Inclusion criteria were an age of 10 to 17 years and a previous diagnosis of high-functioning ASD (defined as full-scale IQ ≥ 85). We decided not to recruit younger children, as our preliminary study revealed that many children below eight years of age are afraid of the android robot because of its realism. Informed consent was obtained from all individual participants and their guardians. IQ eligibility was confirmed within one day of participation using either the Wechsler Intelligence Scale for Children—Third Edition or the Wechsler Adult Intelligence Scale—Third Edition. All participants had previously received a clinical diagnosis of ASD as based on the DSM-5 [21], and this was confirmed through the consensus of a clinical team comprised of experienced professionals (i.e., child and adolescent psychiatrists, clinical psychologists, and pediatric neurologists). The team assessments were made following a detailed clinical examination that took place on the first visit, follow-up observations, and through an evaluation of a questionnaire related to the development and symptoms of participants, which was completed by the guardians. Child and adolescent psychiatrists collected information from guardians concerning developmental milestones (including joint attention, social interaction, pretend play, and repetitive behaviors, with onset prior to 3 years of age) and episodes (e.g., how the individual with ASD behaved at kindergarten and school). Additional professionals, such as teachers and social workers, provided further background information based on their detailed observations of interactions with people (particularly non-family members), repetitive behaviors, obsessive/compulsive traits, and stereotyped behaviors. The sixth author confirmed the existing diagnoses using both diagnostic instruments and screening questionnaires, including the Pervasive Developmental Disorder–Autism Society Japan Rating Scale, which is a diagnostic interview-based scale for ASD developed in Japan [22]. The subscores and total scores of this scale correlate with the domain and total scores of the Autism Diagnostic Interview-Revised [23, 24].

### Procedure

We investigated the preferences of high-functioning individuals with ASD using three types of robots with different appearances: (1) an android robot resembling an adult woman, (2) a mascot robot with a humanoid form and cartoonish appearance, and (3) a mechanical robot with a humanoid form but with many visible mechanical parts. Each participant completed a sequence of three interactional conditions in random order, all of which were guided and facilitated by a family doctor and took place in a standard clinical observation room.

Prior to each trial, the robot was placed on the floor or the table located in the middle of the room. To elicit the belief that the robots were behaving and responding autonomously, we adopted a remote control system similar to those conventionally used in robotics research [25]. Specifically, the robots were operated by researchers who sat in front of a terminal computer located against the wall in the experimental room or in an adjacent room so that they were not visible during the trial. The participants were not informed that the robots were controlled by researchers. The Researchers who operated the robots were different for each participant and each robot. However, each researcher operated a robot according to prepared scripts to make sure that the effects of the difference in operators were negligible.

Participants were individually brought to the room by their caregivers, who remained throughout the entire procedure. Each trial lasted as long as the participants were comfortable in the room, and ended immediately if the children indicated that they wanted to stop the interaction, or if the prepared contents of the interaction had been accomplished after spending at least three minutes in the room. The average duration of each trial was approximately five minutes.

**Fig 1:**
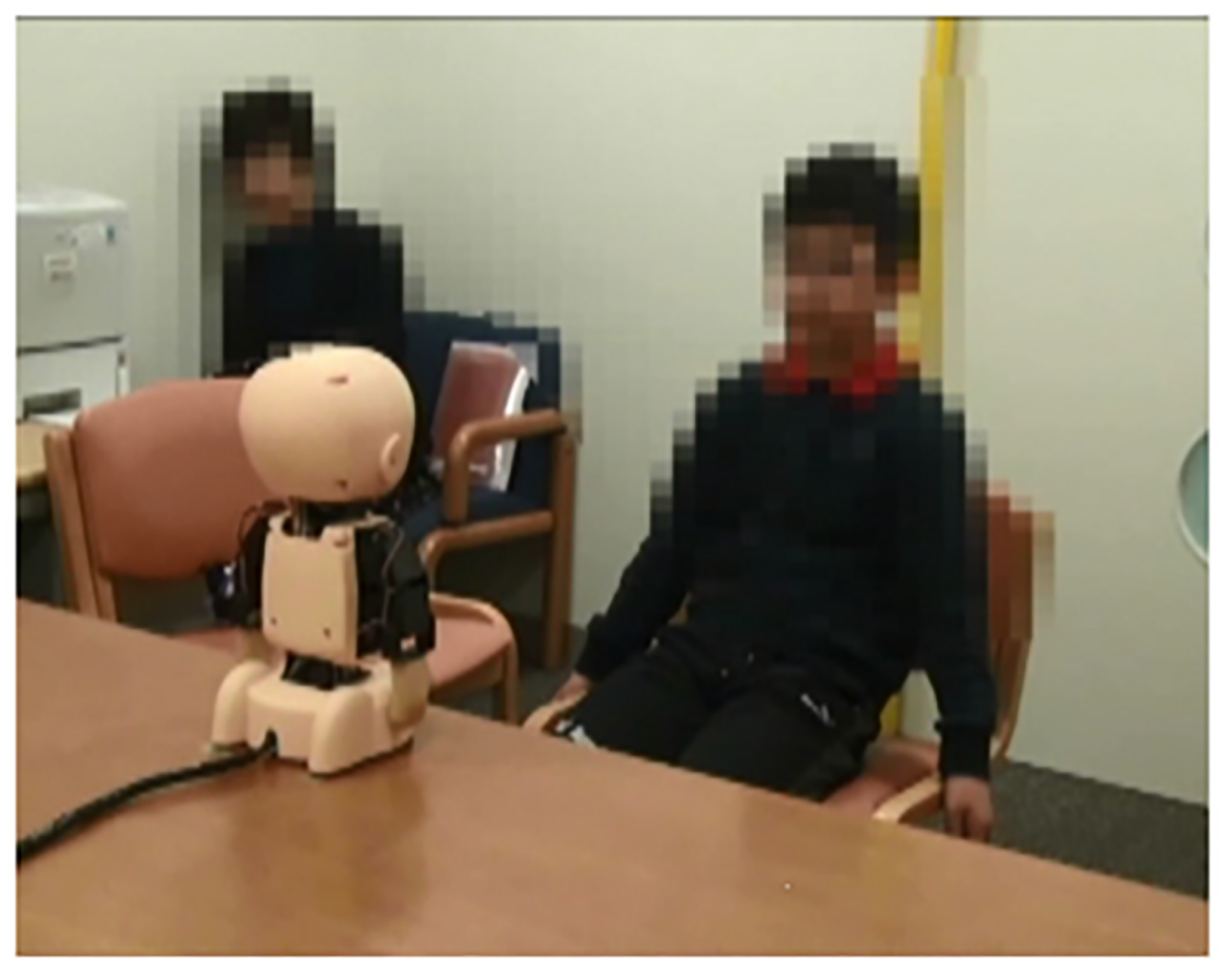
Typical interaction with a robot. The figure shows a participant interacting with the M^3^-synchy.

Fig 1 provides an example of how participants typically interacted with the robots. The experimental rooms had two doors and no windows, and each robot was located in the middle of the room within plain view of the participants. At the conclusion of all the trials, each participant completed a 5-min. semi-structured interview. The interviews were conducted in a familiar room that the children visited often for various activities.

### Questionnaires and interview content

All participants completed the Autism Spectrum Quotient-Japanese version (AQ-J) [26], which was used to assess the presence of milder variants of autistic-like traits to aid in the evaluation of ASD-specific behaviors and symptoms. The AQ-J is a short questionnaire with five subscales (social skills, attention switching, attention to detail, imagination, and communication), and results from this measure have been replicated across both culture [27] and age [28, 29]. The AQ is also sensitive to the broader autism phenotype [30].

In the semi-structured interviews, which were conducted by a human interviewer, the participants were asked three questions about the robots that they had seen. First, the interviewer asked "Do you like robots?" to assess whether each participant liked robots. The interviewer then asked, "Which was your favorite robot," followed by "Which was your second-favorite robot?" These questions enabled us to rank the preferences of the participants. In addition, participants were also encouraged to speak freely about their thoughts on their favorite robot.

### Robots

**Fig 2:**
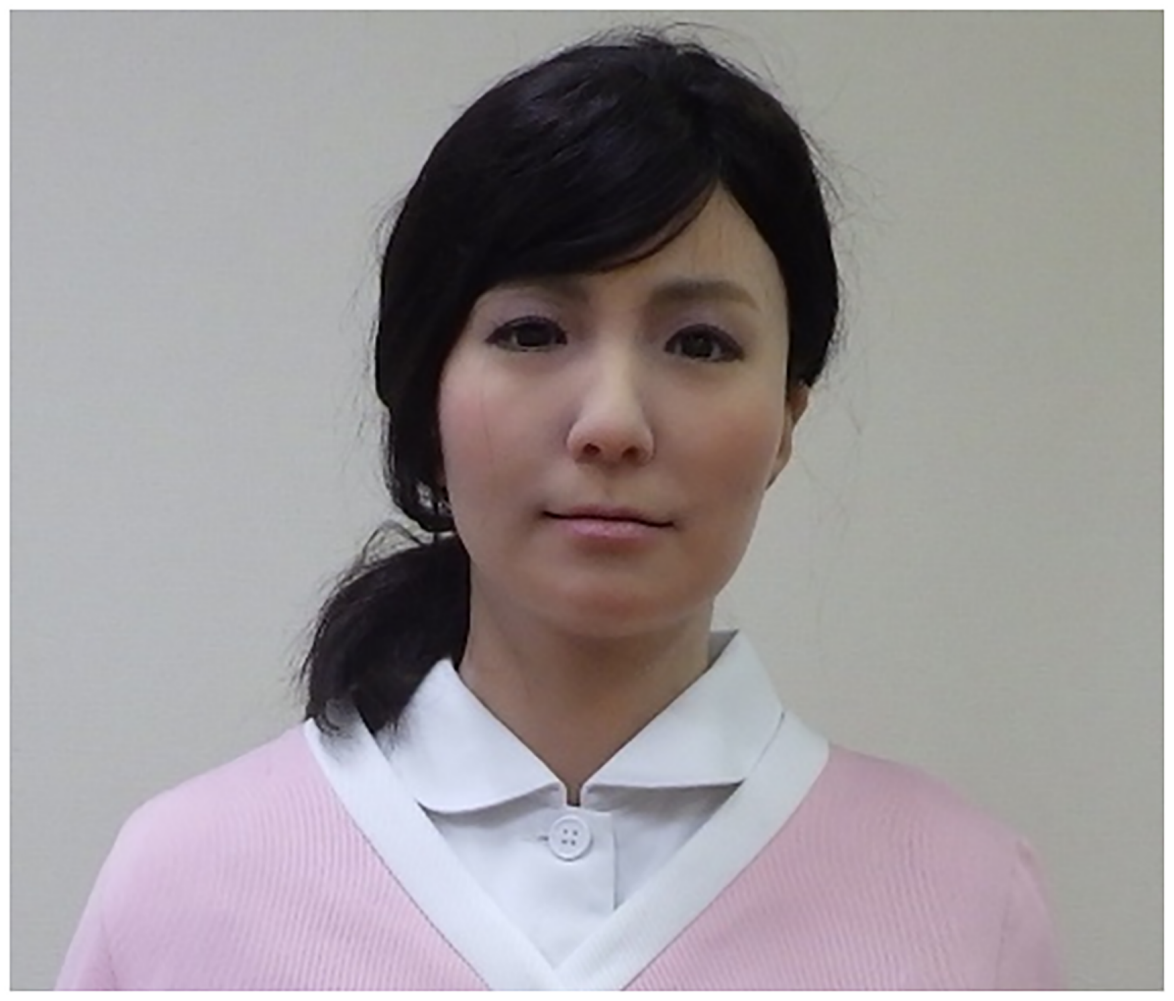
ACTROID-F (android robot).

All robots were tele-operated to perform semi-structured conversations with children. In this experiment, we controlled each robot by implementing the ‘Wizard of Oz paradigm’ to control the effect of human interaction. The robot speech mainly followed a program, and nonverbal interaction was limited. The robot offered a greeting, and then asked the participants questions about how they perceived the robot (e.g., "What am I doing?", "What kind of expression am I making?", "How old do I look?"). Following this, the robot invited the children to touch its chassis. We selected specific robots according to the review by Ricks and Colton [13]. All of the robots had a humanoid form, but the degrees of human likeness were varied as follows:

The android robot used in this study was ACTROID-F (Kokoro Co. Ltd.), which is approximately 165 cm in height and is a female-type humanoid robot with an appearance similar to that of a real person (see Fig 2) [31]. Its artificial body has the same proportions, facial features, hair color, and hairstyle as a human. At first sight, and especially from a distance, it is difficult to distinguish this android robot from an actual human adult.

**Fig 3:**
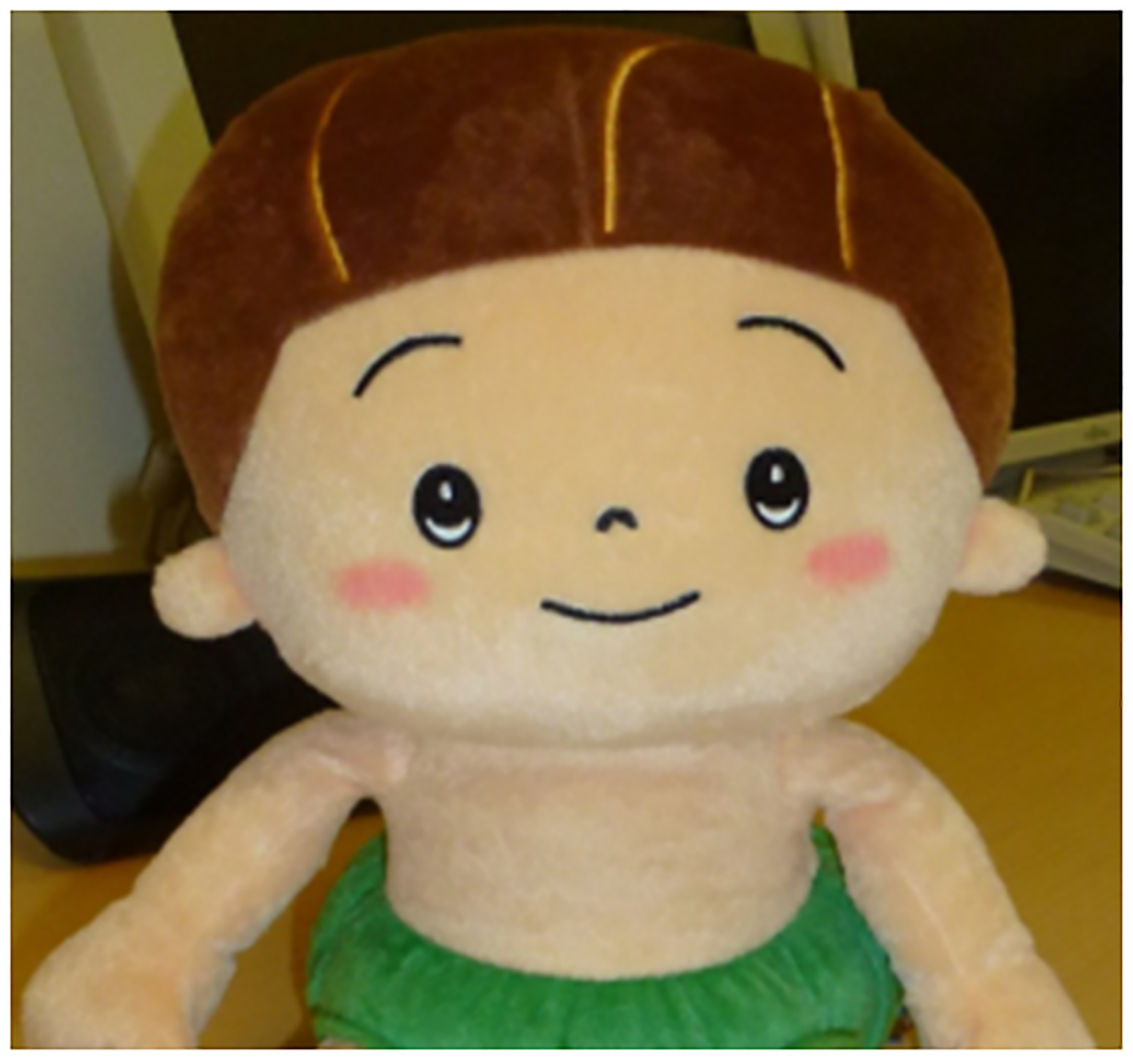
Smile Supplement (mascot robot).

The mascot robot used was a Smile Supplement Robot (PIP Co., Ltd.), which is approximately 28 cm in height and has a humanoid form as well as an abstract or cartoonish appearance [32] (see Fig 3). This robot is dressed in a pumpkin outfit, and has abstracted or exaggerated infant-style features, such as a round symmetrical face and large eyes.

**Fig 4:**
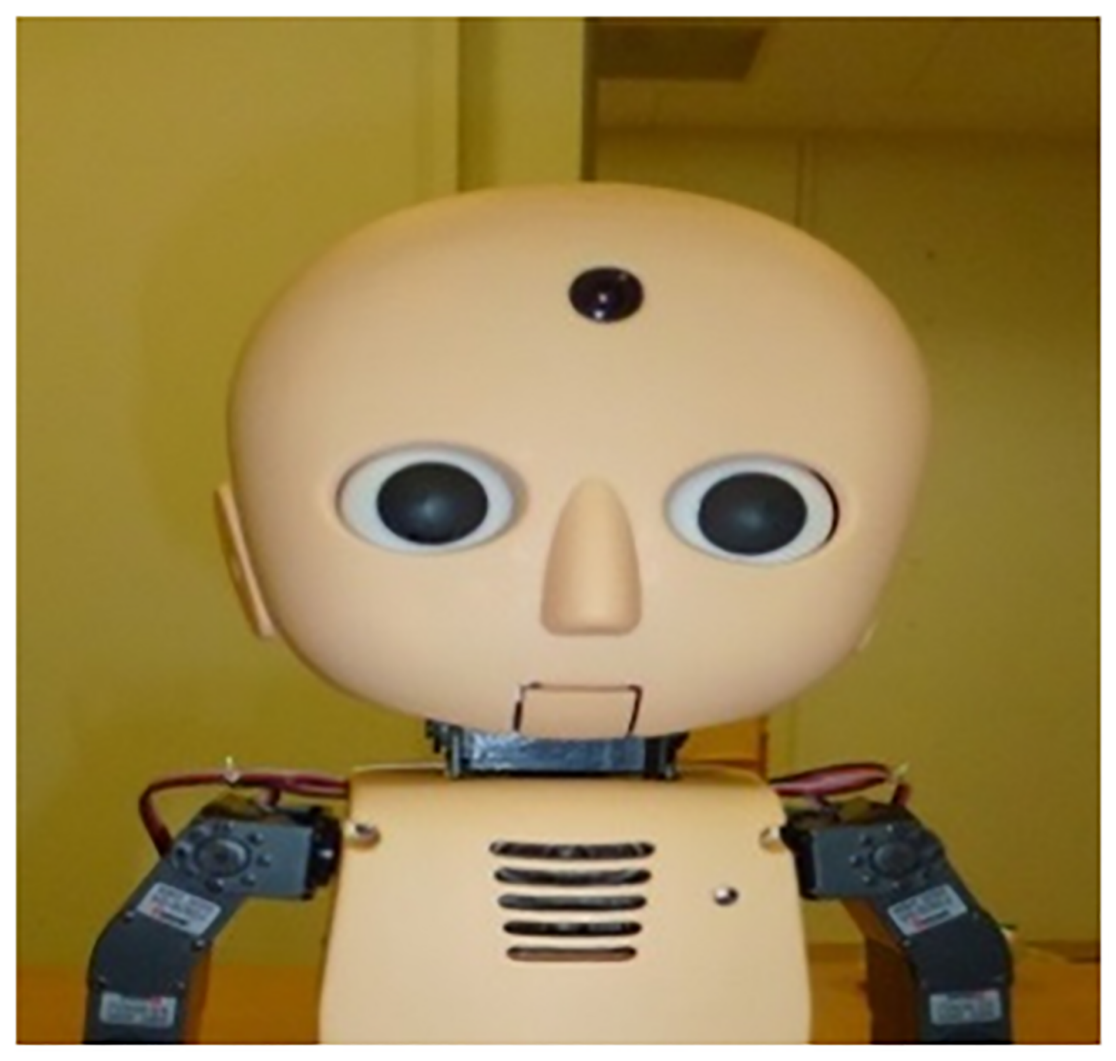
M^3^-synchy (mechanical robot).

The mechanical robot used in this study was an M^3^-Synchy (Vstone Co., Ltd.), which is approximately 30 cm in height and is a child-sized humanoid robot with an abstract appearance and many visible mechanical parts (see Fig 4). The head of this robot has a minimal design, with a focus on features necessary for communication, such as a mouth, eyes, and cheeks, and a small camera on its forehead [33].

### Data analysis

We performed statistical analysis using SPSS version 24.0 (IBM, Armonk, NY, USA). We used descriptive statistics and Spearman’s rank correlation analysis to explore the relationships between robot rank (participant preference), age, AQ-J, PARS scores, and IQ.

## Results

In total, 16 individuals with ASD took part in the study. All participants completed the experimental procedure and the semi-structured interviews. Details are presented in Table 1.

**Table 1:** Descriptive statistics of participants.

Characteristics	N = 16
Age in years	12.6 (2.2)
Gender (Males: Female)	15:1
Full scale IQ	101.1 (7.4)
AQ-J	29.9 (7.4)

AQ-J: autism spectrum quotient, Japanese version. In the AQ-J, higher scores reflect a greater number of ASD-specific behaviors. Parentheses indicate standard deviation

### Interview responses

In response to the first interview question, all 16 participants indicated that they liked robots. When reporting their favorite robot, the android robot was preferred by four participants and selected as the second and third favorite by four and eight participants, respectively. The mascot robot was identified as the favorite by eight participants, with five and three participants ranking it as their second and third favorite, respectively, whereas four participants preferred the mechanical robot, with seven and five participants ranking it second and third, respectively. Two of the cited reasons for android robot favoritism were the advanced technology used to create it, and its resemblance to adults (One of the participants said "I like android robot because it is the most advanced, and it is adult-like."). The mechanical robot was favored for its machine-like appearance (One of the participants said "I like mechanical robot because it is machine-like"). Lastly, the mascot robot was favored for its cuteness and tender appearance (One of the participants said "I like mascot robot because it is cute and seems kind.").

Of the participants, the following observations were made. The participant whose interaction with ACTROID-F was longest in duration favored the Smile Supplement robot the most, and the interaction lasted approximately six minutes. The participant whose interaction with ACTROID-F was shortest in duration favored ACTROID-F the third most, and the interaction lasted approximately three minutes. The participant whose interaction with Smile Supplement robot was longest in duration favored the Smile Supplement robot the most, and the interaction lasted approximately seven minutes. The participant whose interaction with the Smile Supplement robot was the shortest in duration favored the Smile Supplement robot most, and the interaction lasted approximately three minutes. The participant whose interaction with M^3^-synchy was longest in duration favored M^3^-synchy the second most, and the interaction lasted approximately six minutes. The participant whose interaction with M^3^-synchy was the shortest in duration favored M^3^-synchy the second most, and the interaction lasted approximately three minutes.

### Statistical analysis

Table 2 shows the correlations between robot rank and characteristics (age, AQ Total, Full IQ) for the study participants. We found no correlations between robot rank and age or between robot rank and AQ total. Considering age, one of the youngest children (10 years) preferred the mechanical robot, whereas the remaining two children preferred the mascot robot. The oldest children (17 years) preferred the android robot. Conversely, Spearman’s rank correlation analysis revealed significant negative correlations between the AQ-J total score and preference for the android robot (*r* = −0.64, *p* < 0.01). This suggests that there may be important differences in preference that imply that individuals with higher levels of reported ASD symptomatology prefer robots that are more android-like over those perceived as more mechanical or mascot-like. In addition, the IQ of each child who preferred the android robot was at least 100. We found no correlations between the preference ranking of the mascot robot and AQ-J total score, or between the preference ranking of the mechanical robot and AQ-J total score. We also found no correlations between the preference ranking of any robot and the PARS total score and sub-scores for both elementary school and post-junior high school students.

**Table 2:** Correlations: Preference ranking of robot vs. participant demographics.

Preference Ranking of Robot	Age	Full IQ	AQ–J Total
ACTROID-F	-.23	-.34	-.64**
Smile Supplement	.22	.10	.46
M^3^-Synchy	.15	.40	.23

**Significant at p < 0.01

## Discussion

Our results indicate that only four out of 16 high-functioning individuals with ASD preferred to interact with the M^3^-Synchy (mechanical robot) over the other two options. Half of the participants identified the mascot robot as their favorite robot, which is consistent with previous work [13]. Contrary to our hypothesis, our data suggest that there may be important differences in preference, with individuals with higher levels of reported ASD symptomatology preferring the ACTROID-F (android robot), which has an appearance that is more complex than that of the M^3^-Synchy (mechanical robot) or the Smile Supplement Robot (mascot robot). While our sample sizes were somewhat small for statistical comparisons, our quantitative data indicated the above-mentioned trends.

These results are rather surprising, because they challenge the prevailing view that individuals with ASD are intrinsically interested in mechanics. An enormous amount of anecdotal evidence has strongly supported this view, in addition to an influential theory concerning the notion that hyper-systemizing is a feature of the cognitive style of individuals with ASD [34]. Systemizing is the capacity to predict and respond to the behavior of deterministic systems by analyzing input-operation-output relations and inferring the rules that govern such systems. Indeed, this cognitive style is compatible with a preference for mechanics, but the style is related to features such as patterns, rules, regularities, periodicities, etc., that are not restricted to visual appearance. Moreover, these features are more or less shared by all types of robots. Accordingly, one can speculate that robot preference in individuals with ASD is determined based on optimum combinations of each of the above features; this could explain the results of the present study. The study by Robins, Dautenhahn, and Dubowski [18], which suggested that "robots that interact with individuals with ASD should retain a humanoid form", is consistent with the results of our study. Their study is an important point of comparison because they also used the humanoid KASPAR robot, which is designed to look like a human while also being less real than a human. Indeed, our data was consistent with a number of previous studies evaluating the use of "KASPAR" [14, 18–20, 35] for individuals with ASD. However, the high-technology behind the android ACTROID-F may be favored by individuals with ASD, especially those with higher autistic traits. Moreover, a previous study reported that individuals with lower empathy (corresponding to a higher AQ) did not prefer "Baby Schema" [36]. This could justify the use of non-mechanical robots such as FACE, which are designed as an adult android, and to appear as realistic as possible [37].

Medical robots are generally designed with specific therapeutic goals in mind, and these have not previously included preference. However, patient preferences regarding robot appearance and features are crucial to the success of robot-assisted therapy. Individuals with ASD have strong likes and dislikes [38], and if a patient dislikes a therapeutic robot, it may not be possible to perform the therapy. In their guidelines for humanoid robot designs, Ricks and Colton [13] state that individuals with ASD could begin therapy with a simplistic robot, and as they become more comfortable, it may be useful to introduce a more realistic robot. While this approach may be successful, as more highly humanoid robots seem to be effective for the generalization of learned skills, the specific design of the robot should be carefully determined. Thus, sophisticated guidelines may be necessary for the design of therapeutic robots. Our current results may contribute to this process.

We would like to acknowledge several limitations of our study. The first is the relatively small number of participants, the vast majority of whom were male. Although it is common for investigations of humanoid robotics and individuals with ASD to use as few as six participants [13], larger sample sizes are necessary to provide more meaningful data on responses to robotic appearances. In addition, our sample ranged in age from 10 to 17 as our preliminary study revealed that many children below eight years of age are afraid of the android robot because of its realism, whereas the vast majority of research about robots and ASD has focused on a much younger age group. A third limitation is the comparatively short period of interaction between the participants and humanoid robots; however, five minutes per session may be appropriate for the specific characteristics of people with ASD. All participants in this study managed to complete the trial. Fourth, it is important to note that our data concerning humanoid robot preference was based solely on self-report measures and not on direct observation, although many previous studies also rely only on self-report measures [8, 39]. Finally, the apparent gender of the robot also differs across the models tested in the current study; this can have an effect on trust, comfort levels, and preference [40].

## Conclusions

Contrary to our expectations, our data suggest that there may be important differences in preference, with individuals with higher levels of reported ASD symptomatology preferring more human-like humanoid robots over those perceived as more mechanical or mascot-like. Although the general belief regarding individuals with ASD is that they gravitate toward simple, mechanical objects, robot preference may be determined by a variety of factors. The findings of this pilot study meaningfully contribute to research on the influence of robot appearance and provide information about the suitability of specific robot types for therapeutic use.

## Supporting information

S1 File Original data. (XLS)

S1 Video Video of android robot. (WMV)

## References

[1] van der Meer LAJ, Rispoli M. Communication interventions involving speech-generating devices for children with autism: A review of the literature. Developmental Neurorehabilitation. 2010;13(4):294–306. doi: 10.3109/17518421003671494 20629595 10.3109/17518421003671494

[2] Barakova EI, Gillesen JCC, Huskens BEBM, Lourens T. End-user programming architecture facilitates the uptake of robots in social therapies. Robotics and Autonomous Systems. 2013;61(7):704–13. doi: 10.1016/j.robot.2012.08.001

[3] Huskens B, Verschuur R, Gillesen J, Didden R, Barakova E. Promoting question-asking in school-aged children with autism spectrum disorders: Effectiveness of a robot intervention compared to a human-trainer intervention. Developmental Neurorehabilitation. 2013;16(5):345–56. doi: 10.3109/17518423.2012.739212 23586852 10.3109/17518423.2012.739212

[4] Dautenhahn K, Werry I. Towards interactive robots in autism therapy: Background, motivation and challenges. Pragmatics & Cognition. 2004;12(1):1–35. doi: 10.1075/pc.12.1.03dau

[5] Goodwin MS. Enhancing and Accelerating the Pace of Autism Research and Treatment: The Promise of Developing Innovative Technology. Focus on Autism and Other Developmental Disabilities. 2008;23(2):125–8. doi: 10.1177/1088357608316678

[6] Diehl JJ, Schmitt LM, Villano M, Crowell CR. The Clinical Use of Robots for Individuals with Autism Spectrum Disorders: A Critical Review. Res Autism Spectr Disord. 2012;6(1):249–62. doi: 10.1016/j.rasd.2011.05.006 22125579 10.1016/j.rasd.2011.05.006 PMC3223958

[7] Bekele E, Crittendon JA, Swanson A, Sarkar N, Warren ZE. Pilot clinical application of an adaptive robotic system for young children with autism. Autism. 2014;18(5):598–608. doi: 10.1177/1362361313479454 24104517 10.1177/1362361313479454 PMC3980197

[8] Huskens B, Palmen A, Van der Werff M, Lourens T, Barakova E. Improving Collaborative Play Between Children with Autism Spectrum Disorders and Their Siblings: The Effectiveness of a Robot-Mediated Intervention Based on Lego(R) Therapy. J Autism Dev Disord. 2015;45(11):3746–55. doi: 10.1007/s10803-014-2326-0 . 25428293 10.1007/s10803-014-2326-0

[9] Warren ZE, Zheng Z, Swanson AR, Bekele E, Zhang L, Crittendon JA, et al Can Robotic Interaction Improve Joint Attention Skills? J Autism Dev Disord. 2015;45(11):3726–34. doi: 10.1007/s10803-013-1918-4 24014194 10.1007/s10803-013-1918-4 PMC3949684

[10] Scassellati B. How Social Robots Will Help Us to Diagnose, Treat, and Understand Autism. 2007;28:552–63. doi: 10.1007/978-3-540-48113-3_47

[11] Feil-Seifer D, Matarić MJ. Toward Socially Assistive Robotics for Augmenting Interventions for Children with Autism Spectrum Disorders. 2009;54:201–10. doi: 10.1007/978-3-642-00196-3_24

[12] Costescu CA, Vanderborght B, David DO. Reversal Learning Task in Children with Autism Spectrum Disorder: A Robot-Based Approach. J Autism Dev Disord. 2015;45(11):3715–25. doi: 10.1007/s10803-014-2319-z . 25479815 10.1007/s10803-014-2319-z

[13] Ricks DJ, Colton MB. Trends and considerations in robot-assisted autism therapy. 2010:4354–9. doi: 10.1109/robot.2010.5509327

[14] Wainer J, Dautenhahn K, Robins B, Amirabdollahian F. A Pilot Study with a Novel Setup for Collaborative Play of the Humanoid Robot KASPAR with Children with Autism. International Journal of Social Robotics. 2013;6(1):45–65. doi: 10.1007/s12369-013-0195-x

[15] Peca A, Simut R, Pintea S, Costescu C, Vanderborght B. How do typically developing children and children with autism perceive different social robots? Computers in Human Behavior. 2014;41:268–77. doi: 10.1016/j.chb.2014.09.035

[16] Wainer J, Robins B, Amirabdollahian F, Dautenhahn K. Using the Humanoid Robot KASPAR to Autonomously Play Triadic Games and Facilitate Collaborative Play Among Children With Autism. IEEE Transactions on Autonomous Mental Development. 2014;6(3):183–99. doi: 10.1109/tamd.2014.2303116

[17] Bartneck C. Who like androids more: Japanese or US Americans? 2008:553–7. doi: 10.1109/roman.2008.4600724

[18] Robins B, Dautenhahn K, Dubowski J. Does appearance matter in the interaction of children with autism with a humanoid robot? Interaction Studies. 2006;7(3):509–42. doi: 10.1075/is.7.3.16rob

[19] Iacono I, Lehmann H, Marti P, Robins B, Dautenhahn K. Robots as social mediators for children with Autism—A preliminary analysis comparing two different robotic platforms. 2011:1–6. doi: 10.1109/devlrn.2011.6037322

[20] Costa S, Lehmann H, Dautenhahn K, Robins B, Soares F. Using a Humanoid Robot to Elicit Body Awareness and Appropriate Physical Interaction in Children with Autism. International Journal of Social Robotics. 2014;7(2):265–78. doi: 10.1007/s12369-014-0250-2

[21] American Psychiatric Association. (2013). Diagnostic and statistical manual of mental disorders (5th ed). Arlington, VA: American Psychiatric Publishing.

[22] PARS Committee. (2008). Pervasive Developmental Disorders Autism Society Japan Rating Scale. Tokyo: Spectrum Publishing Company.

[23] Lord C, Rutter M, Le Couteur A. Autism Diagnostic Interview-Revised: a revised version of a diagnostic interview for caregivers of individuals with possible pervasive developmental disorders. J Autism Dev Disord. 1994;24(5):659–85. . 7814313 10.1007/BF02172145

[24] Ito H, Tani I, Yukihiro R, Adachi J, Hara K, Ogasawara M, et al Validation of an interview-based rating scale developed in Japan for pervasive developmental disorders. Research in Autism Spectrum Disorders. 2012;6(4):1265–72. doi: 10.1016/j.rasd.2012.04.002

[25] Nishio S, Taura K, Sumioka H, Ishiguro H. Teleoperated Android Robot as Emotion Regulation Media. International Journal of Social Robotics. 2013;5(4):563–73. doi: 10.1007/s12369-013-0201-3

[26] Wakabayashi A, Tojo Y, Baron-Cohen S, Wheelwright S. [The Autism-Spectrum Quotient (AQ) Japanese version: evidence from high-functioning clinical group and normal adults]. Shinrigaku Kenkyu. 2004;75(1):78–84. . 15724518 10.4992/jjpsy.75.78

[27] Wakabayashi A, Baron-Cohen S, Uchiyama T, Yoshida Y, Tojo Y, Kuroda M, et al The autism-spectrum quotient (AQ) children's version in Japan: a cross-cultural comparison. J Autism Dev Disord. 2007;37(3):491–500. doi: 10.1007/s10803-006-0181-3 . 16944324 10.1007/s10803-006-0181-3

[28] Baron-Cohen S, Hoekstra RA, Knickmeyer R, Wheelwright S. The Autism-Spectrum Quotient (AQ)—adolescent version. J Autism Dev Disord. 2006;36(3):343–50. doi: 10.1007/s10803-006-0073-6 . 16552625 10.1007/s10803-006-0073-6

[29] Auyeung B, Baron-Cohen S, Wheelwright S, Allison C. The Autism Spectrum Quotient: Children's Version (AQ-Child). J Autism Dev Disord. 2008;38(7):1230–40. doi: 10.1007/s10803-007-0504-z . 18064550 10.1007/s10803-007-0504-z

[30] Wheelwright S, Auyeung B, Allison C, Baron-Cohen S. Defining the broader, medium and narrow autism phenotype among parents using the Autism Spectrum Quotient (AQ). Mol Autism. 2010;1(1):10 doi: 10.1186/2040-2392-1-10 20678260 10.1186/2040-2392-1-10 PMC2913943

[31] Yoshikawa M, Matsumoto Y, Sumitani M, Ishiguro H. Development of an android robot for psychological support in medical and welfare fields. 2011:2378–83. doi: 10.1109/robio.2011.6181654

[32] Tanaka M, Ishii A, Yamano E, Ogikubo H, Okazaki M, Kamimura K, et al Effect of a human-type communication robot on cognitive function in elderly women living alone. Medical Science Monitor. 2012;18(9):CR550–CR7. doi: 10.12659/MSM.883350 22936190 10.12659/MSM.883350 PMC3560641

[33] Ishiguro H, Minato T, Yoshikawa Y, Asada M. Humanoid Platforms for Cognitive Developmental Robotics. International Journal of Humanoid Robotics. 2011;08(03):391–418. doi: 10.1142/s0219843611002514

[34] Baron-Cohen S. The hyper-systemizing, assortative mating theory of autism. Prog Neuropsychopharmacol Biol Psychiatry. 2006;30(5):865–72. doi: 10.1016/j.pnpbp.2006.01.010 . 16519981 10.1016/j.pnpbp.2006.01.010

[35] Robins B, Dautenhahn K, Dickerson P. From Isolation to Communication: A Case Study Evaluation of Robot Assisted Play for Children with Autism with a Minimally Expressive Humanoid Robot. 2009:205–11. doi: 10.1109/achi.2009.32

[36] Lehmann V, Huis in‘t Veld EMJ, Vingerhoets AJJM. The human and animal baby schema effect: Correlates of individual differences. Behavioural Processes. 2013;94:99–108. doi: 10.1016/j.beproc.2013.01.001 23353724 10.1016/j.beproc.2013.01.001

[37] Pioggia G, Igliozzi R, Sica M L, Ferro M, Muratori F, Ahluwalia A et al Exploring emotional and imitational android-based interactions in autistic spectrum disorders. Journal of Cyber Therapy & Rehabilitation. 2008; 1(1): 49–61.

[38] Trevarthen C, Delafield-Butt JT. Autism as a developmental disorder in intentional movement and affective engagement. Front Integr Neurosci. 2013;7:49 doi: 10.3389/fnint.2013.00049 23882192 10.3389/fnint.2013.00049 PMC3713342

[39] Kaboski JR, Diehl JJ, Beriont J, Crowell CR, Villano M, Wier K, et al Brief Report: A Pilot Summer Robotics Camp to Reduce Social Anxiety and Improve Social/Vocational Skills in Adolescents with ASD. J Autism Dev Disord. 2015;45(12):3862–9. doi: 10.1007/s10803-014-2153-3 . 24898910 10.1007/s10803-014-2153-3

[40] Siegel M, Breazeal C, Norton MI. Persuasive Robotics: The influence of robot gender on human behavior. 2009:2563–8. doi: 10.1109/iros.2009.5354116

